# A Secure and Privacy-Preserving Navigation Scheme Using Spatial Crowdsourcing in Fog-Based VANETs

**DOI:** 10.3390/s17040668

**Published:** 2017-03-24

**Authors:** Lingling Wang, Guozhu Liu, Lijun Sun

**Affiliations:** School of Information Science and Technology, Qingdao University of Science and Technology, Qingdao 266061, China; LGZ_0228@163.com (G.L.); lijunsun@qust.edu.cn (L.S.)

**Keywords:** fog-based VANETs, real-time navigation, privacy-preserving, spatial crowdsourcing

## Abstract

Fog-based VANETs (Vehicular ad hoc networks) is a new paradigm of vehicular ad hoc networks with the advantages of both vehicular cloud and fog computing. Real-time navigation schemes based on fog-based VANETs can promote the scheme performance efficiently. In this paper, we propose a secure and privacy-preserving navigation scheme by using vehicular spatial crowdsourcing based on fog-based VANETs. Fog nodes are used to generate and release the crowdsourcing tasks, and cooperatively find the optimal route according to the real-time traffic information collected by vehicles in their coverage areas. Meanwhile, the vehicle performing the crowdsourcing task can get a reasonable reward. The querying vehicle can retrieve the navigation results from each fog node successively when entering its coverage area, and follow the optimal route to the next fog node until it reaches the desired destination. Our scheme fulfills the security and privacy requirements of authentication, confidentiality and conditional privacy preservation. Some cryptographic primitives, including the Elgamal encryption algorithm, AES, randomized anonymous credentials and group signatures, are adopted to achieve this goal. Finally, we analyze the security and the efficiency of the proposed scheme.

## 1. Introduction

Traffic congestion in crowded urban areas has had a number of negative effects on society, such as wasting motorists’ time, increasing air pollution from the wasted fuel, and creating a higher chance of collisions, etc. It is reported that commuters in Beijing spent on average 32 min per hour in traffic jams while traveling during rush hours in 2015 [1]. Hence, it is a common experience for a driver to find a better driving route in a congested area. Since real-time traffic information plays a key role in monitoring road conditions and predicting optimal routes of vehicles, it is certainly worth using a real-time navigation system for drivers on the road to find the optimal route of a certain destination.

For the last few years, global positioning system (GPS) [2] technology has been adopted for navigation systems, such as the Autonavi navigation system [3], which provides convenient navigation services based on a local map database. However, since the road conditions are not updated in time in the local map database, conventional GPS-based navigation systems may guide drivers to erroneous routes if some traffic accidents occur in real time.

In the meantime, vehicular ad hoc networks (VANETs), which act as important elements of the intelligent transportation system, has become increasingly popular in many countries. The navigation system based on VANETs can provide more timely and more accurate traffic information. In a typical VANET, vehicles are equipped with on-board units (OBUs) to perform mobile computation and communication with other nearby vehicles, and with road-side units (RSUs) installed along the road. With the support of VANETs and its crowdsensing capability, real-time road conditions can be collected and transmitted to support the real-time navigation. However, RSUs have limited computation and storage capability, while real-time navigation systems based on crowd sourcing require complex computation and large storage. This challenge has motivated researchers to investigate the new paradigm of VANETs.

Recently, the vehicular cloud has been proposed for big data processing and complicated intelligent analysis on VANET environments [4,5,6]. However, centralized cloud computing is unnecessary and inefficient for the interactive navigation system. To relieve the computation and communication burden on vehicular cloud, fog computing can be adopted. Fog computing, which was firstly proposed by Cisco in 2012 [7], is an extension of the cloud-based Internet by introducing an intermediate layer between mobile devices and cloud, aiming at the smooth, low-latency service delivery from the cloud to mobile. In this paper, we combine vehicular cloud with fog computing, and consider a new paradigm called fog-based VANETs.

Although fog-based VANETs can provide many possible advantages for real-time navigation systems, several security concerns also have to be addressed before the implementation of the system. A navigation system requires that the identity of the vehicle and communication messages should be authenticated and maintain secrecy to guard against the impersonation, message forgery attacks, and eavesdropping. Meanwhile, privacy preservation must be achieved because the private information, such as vehicles’ licenses, location, and speed, etc., needs to be protected. In addition, the authorities should be able to reveal the real identity of the disputed vehicles when necessary.

In this paper, we proposed a secure and privacy-preserving navigation scheme (SPNS) in fog-based VANETs, which use spatial crowdsourcing to collect real-time traffic information and analyze the collected data to provide real-time navigation services to drivers. The security and the privacy preservation of the scheme are also analyzed to evaluate the scheme. In particular, we make the following contributions:

- First, we present a model for a secure navigation scheme in fog-based VANETs, which takes advantage of vehicular cloud and fog computing to make up for the limitation of the previous VANET-based navigation system.
- Second, we construct a specific scheme that can support real-time navigation service to drivers in a congested area. In this way, drivers can quickly find an available route, and, moreover, gasoline and the time wasted in traffic congestion can be reduced. By using the spatial crowdsourcing, the real-time road conditions can be updated in time in fog-based VANETs. Meanwhile, the vehicle performing the crowdsourcing task can get a reasonable reward. Performance analysis shows that the real-time navigation service supported by the proposed scheme is effective.
- Third, the proposed scheme can also ensure the conditional privacy preservation of the vehicles (or drivers), which is regarded as the basic security requirement in VANET communications [8,9,10,11].

The rest of the paper is organized as follows: the system model and design goals are described in Section 2. Some preliminaries are given in Section 3. Our scheme is proposed in Section 4. The security analysis is given in Section 5, and the performance analysis is given in Section 6. Related work is reviewed in Section 7. Finally, Section 8 concludes the paper.

## 2. System Model and Design Goal

In this section, we define the problem by formalizing the system model and the design goal.

### 2.1. System Model

**Figure 1:**
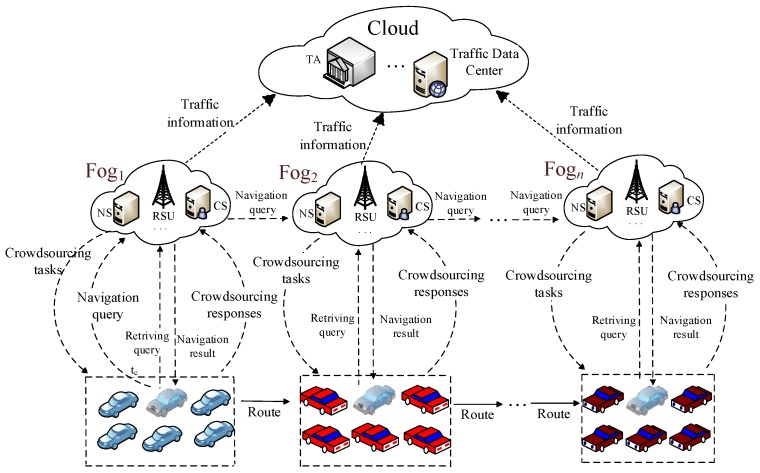
System model.

In this section, we consider the fog-based VANETs and describe our system model, in which communication nodes include trusted authority (TA), navigation servers (NS) and crowdsourcing servers (CS) residing in the fog, and vehicles as shown in Figure 1. The detailed description of system components is as follows:

- TA is trusted and the public agency. For example, the transportation authorities with administrative rights can take on the role of the TA. It is responsible for the registration of fog nodes and vehicles deployed in fog-based VANETs, issuing anonymous credentials and tracing the vehicles’ identity in case of rewarding purpose for spatial crowdsourcing, sending fake traffic information for uncongested driving experience, etc.
- The cloud is a set of interconnected computing resources. The cloud provides centralized navigation services for drivers, e.g., Google Map.
- Fog node is a highly virtualized computing system, which is deployed at the edge of networks, e.g., banks, bus terminals, shopping halls, etc. Similar to a lightweight cloud server, fog node is equipped with the on-board large volume data storage, computers and wireless communication facility [12]. In our fog-based VANETs, the fog node consists of navigation servers (NS) and crowdsourcing servers (CS) and conventional RSU, which are in charge of releasing crowdsourcing tasks, computing the optimal path for the querying vehicles, and rewarding the crowdsourcing contributors.
- Vehicles are equipped with irreplaceable and temper-proof OBU device, which enables performing some simple computations, communicating with other vehicles and fog nodes, and these vehicles have a small amount of read-only memory. In our model, OBU is required to generate real-time navigation query, traffic information reports for spatial crowdsourcing tasks, result retrieving query.

As shown in Figure 1, the navigation scheme works as the following. Assume each vehicle and fog node have already registered to the TA. Then, a vehicle can send a navigation query generated by the OBU to the nearby fog node, denoted as fog${}_{1}$, at time $t_{0}$. The navigation server (NS) in Fog${}_{1}$ forwards the query to the last Fog${}_{n}$ which covers the destination by relaying fog nodes hop by hop. Upon receiving the navigation query, each crowdsourcing server (CS) generates and releases a crowdsourcing task of collecting real-time traffic information to vehicles in its coverage area. In addition, the vehicle who wants to perform the task returns a crowdsourcing report and can get a reasonable reward from the CS. Upon receiving the report, CS verifies it and shares the valuable traffic information with NS. The NS computes the optimal path for the querying vehicle in its area. Meanwhile, NS analyzes and forwards the traffic information to the cloud for other services. Finally, the querying vehicle can get successive navigation results from fog nodes by sending navigation result retrieving query when entering the coverage of the fog nodes until it ultimately reaches the desired destination.

### 2.2. Design Goal

Before describing our design goal for the navigation scheme, we first make necessary assumptions in our system model.

**Assumption** **1.** TA is fully trusted by all vehicles and fog nodes. TA can communicate with fog nodes and vehicles through a secure channel by the internet or any other reliable communication links with high bandwidth. The TA can also inspect all fog nodes and maintain the compromised entities list.

**Assumption** **2.** Fog nodes are untrusted. For instance, some honest-but-curious fog nodes may learn the position of specific vehicles and also get some sensitive information for some purposes.

**Assumption** **3.** The adversary can overhear V2V (Vehicle to Vehicle) and V2I (Vehicle to Infrastructure) communications to obtain any messages for their purposes, such as tracing the identity of some vehicles. Some dishonest vehicles may overhear the communications to obtain the navigation results queried by other vehicles to enjoy free navigation services if they happen to have the same destination.

**Assumption** **4.** *TA, fog nodes, and vehicles have clocks for generation of time stamps and check valid time of navigation query and result retrieving query. They can use GPS satellites as a synchronized time source* [13].

Our design goal is to develop a secure and privacy-preserving navigation scheme for vehicles, which can achieve the following desirable requirements: (1) real-time route navigation; (2) authentication; (3) confidentiality; and (4) conditional privacy preservation.

- Real-time path navigation: With the guidance of the fog nodes, a vehicle can conveniently find the optimal path to the desired destination.
- Authentication: Only legitimate entities should take part in the fog-based VANETs. Fog nodes and vehicles should be able to prove themselves by using certificates or credentials. In addition, the origin of the messages should be authenticated to prevent against the impersonation and message forgery attacks. Meanwhile, the identity of the crowdsourcing contributor should be authenticated to get the reward. In addition, only a legitimate subscriber that has service access rights should be able to get navigation service.
- Confidentiality: the navigation query, traffic information report, and navigation result should be kept confidential from eavesdroppers who will illegally use the navigation information for their own purposes.
- Conditional privacy preservation: the real identity of the querying vehicle and the crowdsourcing vehicle should be kept secret. Although the location and destination would be exposed to fog nodes, the adversary can neither link a navigaiton query to a specific vehicle nor identify two navigation queries from the same vehicle. However, once an exceptional event occurs, the fog nodes can learn the vehicles’ real identifier with the help of TA.

## 3. Preliminaries

This section describes some cryptographic primitives which are adopted in our proposed scheme. They are bilinear groups, message-locked encryption, randomized signatures and group signatures.

### 3.1. Bilinear groups

Bilinear groups are a set of three cyclic groups $G_{1}$, $G_{2}$, $G_{T}$ of prime order *q* with a bilinear map $e:G_{1}\times G_{2}\to G_{T}$ with the following properties:

1. for all $g_{1}\in G_{1}$, $g_{2}\in G_{2}$ and $a,b\in Z_{q}$, $e\left(g_{1}^{a},g_{2}^{b}\right)=e{\left(g_{1},g_{2}\right)}^{ab}$;
2. for $g_{1}\neq 1_{G_{1}}$ and $g_{2}\neq 1_{G_{2}}$, $e\left(g_{1},g_{2}\right)\neq 1_{G_{T}}$ ;
3. the map *e* is efficiently computable.

There are three types of pairings defined by Galbraith, Paterson, and Smart [14]: in type 1, $G_{1}=G_{2}$; in type 2, $G_{1}\neq G_{2}$, but there exists an efficient homomorphism $\phi :G_{2}\to G_{1}$ while no efficient one exists in the other direction; in type 3, $G_{1}\neq G_{2}$ and there is no efficiently computable homomorphism between $G_{1}$ and $G_{2}$ in either direction. In this paper, we only consider type 3 pairings, which will guarantee the security of the randomized signatures used in our scheme.

### 3.2. Message-Locked Encryption

A message-locked encryption (MLE) scheme is a symmetric encryption scheme in which the key used for encryption and decryption is itself derived from the message [15]. Instances of this primitive are seeing widespread deployment and application for the purpose of secure deduplication [16,17,18]. A message-locked encryption scheme MLE = (P,K,E,D,T) is a five-tuple of polynomial time algorithm, the last two deterministic:

- On input $1^{\lambda }$, the parameter generation algorithm P returns a public parameter *P*.
- On input *P* and a message *M*, the key-generation algorithm K returns a message-derived key $K\leftarrow K_{P}\left(M\right)$.
- On input P,K,M, the encryption algorithm E returns a ciphertext $C\leftarrow E_{P}\left(K,M\right)$.
- On input P,K and a ciphertext *C*, the decryption algorithm D returns $D_{P}\left(K,C\right)\in {\left\{0,1\right\}}^{*}\cup \left\{⊥\right\}$.
- On input P,C, the tag generation algorithm returns a tag $T\leftarrow T_{P}\left(C\right)$.

Bellare [15] has summarized four MLE schemes: convergent encryption (CE), Hash-and-CE1 (HCE1), Hash-and-CE2 (HCE2), and randomized convergent encryption (RCE). In our scheme, we utilize the RCE to encrypt the crowdsourcing traffic information report.

### 3.3. Randomized Signatures

Randomized signature [19] is both an efficient and secure signature with the same features as Camenisch-Lysyanskaya (CL)-signatures [20] but consists of only two elements in the signature. It takes advantage of the full potential of type 3 pairings, in which the space of the signatures and the one of the public key are seperated. A randomized signature scheme usually consists of four algorithms:

- $Setup(1^{k})$: given a security parameter *k*, this algorithm outputs public parameter $param=(q,G_{1},G_{2},G_{T},e)$. These bilinear groups must be of type 3.
- Keygen(param): selects $g_{2}\in G_{2}$ and $\left(x_{1},x_{2}\right)\in Z_{q}^{2}$, computes $\left(X_{1},X_{2}\right)=\left(g_{2}^{x_{1}},g_{2}^{x_{2}}\right)$, and sets sk as $\left(x_{1},x_{2}\right)$ and pk as $\left(g_{2},X_{1},X_{2}\right)$.
- Sign(sk,m): picks a random $g_{1}\in G_{1}^{*}$ to compute a signature $\sigma \leftarrow (g,g^{x_{1}+mx_{2}})$.
- Verify(σ,pk,m): parses σ as $\sigma_{1},\sigma_{2}$ and checks whether $\sigma_{1}\neq 1_{G_{1}}$ and $e\left(\sigma_{1},X_{1}X_{2}^{m}\right)=e\left(\sigma_{2},g_{2}\right)$ are both satisfied. If it is the positive case, it outputs 1, and 0 otherwise.

A randomized signature $\left(\sigma_{1},\sigma_{2}\right)$ on *m* can be randomized by selecting a random $r\in Z_{q}$ and computing $\sigma^{\prime }\leftarrow \left(\sigma_{1}^{r},\sigma_{2}^{r}\right)$ , which is still a valid signature on *m*.

### 3.4. Group Signatures

The group signature scheme was first introduced by David Chaum and Eugene van Heyst in 1991 [21]. In a group signature scheme, there exists a group manager and several group members, essential to which is a group manager, who is in charge of adding group members and has the ability to reveal the original signer in the event of disputes. A group signature scheme is desired to satisfy three security properties: unforgeability, anonymity, and traceability. Unforgeability ensures that only the group member can generate signatures on behalf of the group. Anonymity means that signatures do not reveal their signer’s identity, except the group manager. Traceability shows that all valid signatures, even those generated by the collusion of multiple group members, can be revoked by the group manager.

## 4. Proposed Secure and Privacy-Preserving Navigation Scheme

In this section, we present a secure and privacy-preserving navigation scheme (SPNS) in fog-based VANETs, which consists of four parts: (1) system setting; (2) real-time navigation querying; (3) vehicular spatial crowdsourcing; and (4) navigation result retrieving.

### 4.1. System Setting

Let $G_{1},G_{2}$, and $G_{T}$ be three cyclic groups of the same large prime order *q*. Suppose that $G_{1},G_{2}$, and $G_{T}$ are equipped with a type 3 pairing. Let $H:{\left\{0,1\right\}}^{*}\to Z_{q}^{*}$ be a public collision-resistant hash function. The TA first chooses $\left(x_{1},x_{2}\right)\in Z_{q}^{2}$ as the master key and computes $\left(X_{1},X_{2},X_{3},X_{4}\right)=\left(g_{1}^{x_{1}},g_{2}^{x_{2}},g_{1}^{x_{2}},g_{2}^{x_{1}}\right)$ as its public key. In the end, the TA publishes the system parameters $P\;=\;(q,G_{1},G_{2},G_{T},e,g_{1},g_{2},X_{1},X_{2},X_{3},X_{4})$.

Each fog node has a unique identifier $R_{ID}$ to identify its position and a map of its coverage. $R_{ID}$ randomly chooses $y\in Z_{q}$ as its secret key and computes $Y=g_{2}^{y}$ as its public key. In addition, $R_{ID}$ maintains a routing table to determine the next fog node to which the vehicle should move forward.

Each vehicle has a unique identifier $V_{ID}$. The vehicle $V_{ID}$ randomly chooses $v\in Z_{q}$ to compute $V=g_{1}^{v}$, $V_{1}=X_{2}^{v}$, and sends $\left(V,V_{1}\right)$ to the TA to prove its knowledge of *v*. Then, the TA verifies the validity by checking the equation $e\left(V,X_{2}\right)=e\left(g_{1},V_{1}\right)$. If the equation does not hold, the TA returns failure and aborts. Otherwise, the TA picks $s\in Z_{q}$ to compute

$$\left(A,A_{1},A_{2}\right)\leftarrow \left(g_{1}^{\frac{1}{x_{1}+s}},g_{1}^{s},{\left(X_{1}\cdot V^{x_{2}}\right)}^{s}\right).$$

In addition, the TA stores $\left(V_{ID},A\right)$ in a secure database, and returns $\left(A,A_{1},A_{2}\right)$ to $V_{ID}$ through a secure channel. $R_{ID}$ sets its secret key as $skv=(v,A,A_{1},A_{2})$ and the corresponding public key as pkv=V.

When $V_{ID}$ starts to travel in the city, it will generate some short-life keys for navigation queries according to the following steps:

- $V_{ID}$ chooses *m* random numbers, $u_{1},u_{2},\cdots ,u_{m}\in Z_{q}^{*}$ as the short-life private keys and computes the corresponding public keys $U_{l}=g_{2}^{u_{l}}$ for l=1,2,⋯,m for the travel;
- for each short-life public key $U_{l}$, $V_{ID}$ computes the self-delegated certificate $Cert_{l}$ as follows:
  − randomly choose α, $t_{\alpha }$, $t_{v}$, $t_{\beta }\in Z_{q}$, compute $T_{1},T_{2},\beta ,\beta_{1},\beta_{2},\beta_{3}$ as follows: $T_{1}=X_{1}^{\alpha }$, $T_{2}=A\cdot X_{3}^{\alpha }$, β=α·v mod *q*, $\beta_{1}=X_{1}^{t_{\alpha }}$, $\beta_{2}=T_{1}^{v}/X_{1}^{t_{\beta }}$, $\beta_{3}=e\left(T_{2},g_{2}^{t_{v}}\right)\cdot e\left(X_{3},X_{4}^{t_{\alpha }\cdot g_{2}^{t_{\beta }}}\right)$;
  − compute $c=H(X_{1},X_{3},U_{l},T_{1},T_{2},\beta_{1},\beta_{2},\beta_{3})$ and $s_{\alpha },s_{v},s_{\beta }$ where $s_{\alpha }=t_{\alpha }+c\cdot \alpha$ mod *q*; $s_{v}=t_{v}+c\cdot v$ mod *q*; $s_{\beta }=t_{\beta }+c\cdot \beta$ mod *q*; and the certificate of $U_{l}$ is $Cert_{l}=\left\{U_{l},T_{1},T_{2},c,s_{\alpha },s_{v},s_{\beta }\right\}$.
  − anyone can check the validity of $U_{l}\left|\right|Cert_{l}$ by computing: $\beta_{1}^{\prime }=X_{1}^{s_{\alpha }}/T_{1}^{c}$;
    $\beta_{2}^{\prime }=T_{1}^{s_{v}}/X_{1}^{s_{\beta }}$;
    $\beta_{3}^{\prime }=e\left(T_{2},g_{2}^{s_{v}}\cdot X_{4}^{c}\right)/e\left(X_{3},X_{4}^{s_{\alpha }}\cdot g_{2}^{s_{\beta }}\right)e\left(g_{1},g_{2}^{c}\right)$;
    and check whether $c=H(X_{1},X_{3},U_{l},T_{1},T_{2},\beta_{1}^{\prime },\beta_{2}^{\prime },\beta_{3}^{\prime })$ holds.
- $V_{ID}$ installs skv and $u_{l}\left|\right|U_{l}\left|\right|Cert_{l}$ for l=1,2,⋯,m into the read-only memory of the OBUs.

### 4.2. Real-Time Navigation Querying

When a vehicle $V_{ID}^{*}$ that is equipped with an OBU is driving on the road, it can send a real-time navigation query to the nearby fog node, denoted as $R_{1}$. The real-time query utilizes the OBU to generate the navigation information {N, $U^{*}$, CL, DEST, $t_{c}$, $t_{e}\}$, as shown in Table 1.

Using this navigation information, the querier $V_{ID}^{*}$ performs the following steps to generate a navigation query:

- utilize $Y_{1}$ to encrypt $\left(U^{*},CL,DEST\right)$ by randomly choosing $k_{1}\in Z_{q},g_{0}\in G_{1}$, and compute $C_{1}=g_{1}^{k_{1}}$, $C_{2}=g_{0}\cdot Y_{1}^{k_{1}}$, $C_{3}=AES_{Enc}(g_{0},U^{*}\left||CL||DEST\right)$;
- select randomly $\left(r_{1},r_{2}\right)\in Z_{q}^{2}$ to compute the randomized signture $\left(B_{1},B_{2}\right)\leftarrow \left(A_{1}^{r_{1}},A_{2}^{r_{1}}\right)$, and the hush function $c=H(B_{1},B_{2},e{\left(B_{1},X_{2}\right)}^{r_{2}},N,U^{*},CL,DEST,t_{c},t_{e})$, $\tau =r_{2}+c\cdot v$, and output the group signature $\left(B_{1},B_{2},c,\tau \right)$;
- finally, send the navigation query *Q* to the fog node $R_{1}$, where $Q=(N,t_{c},t_{e},C_{1},C_{2},C_{3},B_{1},B_{2},c,\tau )$.

Upon receiving the navigation query *Q*, $R_{1}$ firstly checks whether the destination DEST is in its coverage. If the answer is yes, it will generate a crowdsourcing task to find the optimal route to the destination for the querying vehicle $V_{ID}^{*}$. Otherwise, $R_{1}$ performs the following steps to forward *Q* to the next fog node $R_{2}$ according to its routing table. Meanwhile, it will generate a crowdsourcing task to find the optimal route to the next fog node for the querying vehicle.

- Decode $\left(U^{*},CL,DEST\right)=AES_{Dec}\left(g_{0},C_{3}\right)$ by computing $g_{0}=C_{2}\cdot C_{1}^{-y_{1}}$;
- verify the validity of the signature $\left(B_{1},B_{2},c,\tau \right)$ by computing
  $$B=e\left(B_{1},X_{1}^{c}\right)\cdot e\left(B_{2},g_{2}^{-c}\right)\cdot e\left(B_{1},X_{2}^{\tau }\right)$$
  and checking whether the hash $c=H(B_{1},B_{2},B,N,U^{*},CL,DEST,t_{c},t_{e})$ holds. If not, $R_{1}$ returns failure and aborts; Otherwise, it checks the routing table to find the next fog node, denoted as $R_{2}$, according to the destination DEST.
- $R_{1}$ randomly chooses $k_{1}^{\prime }\in Z_{q},g_{0}^{\prime }\in G_{1}$ to compute $C_{1}^{\prime }=g_{1}^{k_{1}^{\prime }}$, $C_{2}^{\prime }=g_{0}^{\prime }\cdot Y_{2}^{k_{1}^{\prime }}$, $C_{3}^{\prime }=AES_{Enc}(g_{0}^{\prime },U^{*}\left||CL||DEST\right)$;
- finally, $R_{1}$ forwards the query $Q^{\prime }=\left(N,t_{c},t_{e},C_{1}^{\prime },C_{2}^{\prime },C_{3}^{\prime },B_{1},B_{2},c,\tau \right)$ to $R_{2}$.

When $R_{2}$ receives $Q^{\prime }$, it performs the same operations as $R_{1}$ and will forward the query to the next fog node until it reaches the last fog node, denoted as $R_{n}$, which covers the destination of the querying vehicle $V_{ID}^{*}$.

### 4.3. Spatial Crowdsourcing

When receiving a navigation query with a sequence number *N*, the fog node $R_{j}\;\in \;\left\{R_{1},R_{2},\cdots ,R_{n}\right\}$ generates and releases a crowdsourcing task of collecting traffic information to all the vehicles in its coverage area. $R_{j}$ keeps a tag table TT, which contains all tags of rewarded vehicles for a specific crowdsourcing task. If a vehicle $V_{ID}^{i}$ with the secret key $skv_{i}=\left(v_{i},A_{i},A_{1i},A_{2i}\right)$ wants to perform this task, it performs the operations as follows:

- randomly choose a short-life public key $U_{i}$ from the $u_{l}\left|\right|U_{l}\left|\right|Cert_{l}$ for l=1,2,⋯,m stored in its OBU;
- generate a traffic information report $P_{i}$ including the current location, current time, driving speed and the road condition;
- randomly choose $\left(r_{1i},r_{2i}\right)\in Z_{q}^{2}$ to calculate the randomized signature $\left(B_{1i},B_{2i}\right)\leftarrow \left(A_{1i}^{r_{1i}},A_{2i}^{r_{2i}}\right)$, the hash $c_{i}=H\left(N,P_{i},B_{1i},B_{2i},e{\left(B_{1i},X_{2}\right)}^{r_{2i}}\right)$, and $\tau_{i}=r_{2i}+c_{i}\cdot v_{i}$;
- encrypt the traffic information report $P_{i}$ by using a message-lock encryption algorithm. Choose $L_{i}\in Z_{q}^{*}$ to compute $K_{i}=H\left(P,P_{i}\right)$, the tag $T_{i}=H\left(P,K_{i}\right)$, $E_{i}=AES_{Enc}\left(L_{i},P_{i}\right)$, and $H_{i}\;=\;L_{i}⊕K_{i}⊕U_{i}$;
- finally, $V_{ID}^{i}$ returns the crowdsourcing response $RP_{i}=\left(N,U_{i},T_{i},E_{i},H_{i},B_{1i},B_{2i},c_{i},\tau_{i}\right)$ to $R_{j}$.

When $R_{j}$ receives the crowdsourcing response $RP_{i}$, it will compute the optimal path to the destination by the following operations:

- decode the crowdsourcing response $P_{i}$ by computing $L_{i}=H_{i}⊕K_{i}⊕U_{i}$, $P_{i}=AES_{Dec}\left(L_{i},E_{i}\right)$, and the tag $T_{i}^{\prime }=H\left(P,H\left(P,P_{i}\right)\right)$;
- check whether the equation $T_{i}^{\prime }=T_{i}$ holds. If not, $R_{j}$ reject the $RP_{i}$. Otherwise, it compares the tag $T_{i}$ with the element in the tag table TT. If there exists a tag $T_{j}$ in the tag table TT satisfying $T_{i}=T_{j}$, which means the same traffic information report has been stored in the database, $R_{j}$ will reject the $RP_{i}$. Otherwise, it verifies the signature by computing
  $$B_{i}=e\left(B_{1i},X_{1}^{c_{i}}\right)\cdot e\left(B_{2i},g_{2}^{-c_{i}}\right)\cdot e\left(B_{1i},X_{2}^{\tau_{i}}\right)$$
  and checking whether the equation $c_{i}=H\left(N,P_{i},B_{1i},B_{2i},B_{i}\right)$ holds. If not, $R_{j}$ returns failure and aborts; otherwise, it keeps $RP_{i}$ in its database;
- $R_{j}$ rewards the contributor $V_{ID}^{i}$ based on the short-life public key $U_{i}$;
- $R_{j}$ can compute the optimal path $OP_{j}$ by using Dijkstra’s algorithm in its coverage area;
- choose $k_{1i}\in Z_{q}$, $g_{0i}\in G_{2}$ to calculate $\left(e_{1i},e_{2i},e_{3i}\right)=\left(g_{2}^{k_{1i}},g_{0i}\cdot U^{*k_{1i}},AES_{Enc}\left(g_{0i},OP_{j}\right)\right)$, and $S_{j}=U^{*y_{j}}$. Finally, the navigation result for $V_{ID}^{*}$ is $\left(N,S_{j},e_{1i},e_{2i},e_{3i}\right)$.

### 4.4. Navigation Result Retrieval

When the querying vehicle $V_{ID}^{*}$ enters the coverage area of $R_{j}$, it reads $\left(u^{*},U^{*}\right)$ in the OBU, computes $S_{j}^{\prime }=Y_{j}^{u^{*}}$ and generates the retrieving query RQ.

$V_{ID}^{*}$ chooses randomly $\left(r_{1}^{\prime },r_{2}^{\prime }\right)\in Z_{q}^{2}$ to compute randomized signature $\left(B_{1}^{\prime },B_{2}^{\prime }\right)\leftarrow \left(A_{1}^{r_{1}^{\prime }},A_{2}^{r_{1}^{\prime }}\right)$, the hash value $c^{\prime }=H\left(t_{c},S_{j}^{\prime },B_{1}^{\prime },B_{2}^{\prime },e{\left(B_{1}^{\prime },X_{2}\right)}^{r_{2}^{\prime }}\right)$, in which $t_{c}$ is the current time. $\tau^{\prime }=r_{2}^{\prime }+c^{\prime }\cdot v^{\prime }$, and sends $RQ=(t_{c},S_{j}^{\prime },B_{1}^{\prime },B_{2}^{\prime },c^{\prime },\tau^{\prime })$ to the fog node $R_{j}$.

Upon receiving RQ, $R_{j}$ computes

$$B^{\prime }=e\left(B_{1}^{\prime },X_{1}^{c^{\prime }}\right)\cdot e\left(B_{2}^{\prime },g_{2}^{-c^{\prime }}\right)\cdot e\left(B_{1}^{\prime },X_{2}^{\tau^{\prime }}\right)$$

and checks whether the equation $c^{\prime }=H\left(t_{c},S_{j}^{\prime },B_{1}^{\prime },B_{2}^{\prime },B^{\prime }\right)$ holds. If not, $R_{j}$ returns failure and aborts; otherwise, it searches for the navigation result $\left(N,S_{j},e_{1i},e_{2i},e_{3i}\right)$ in the database based on $S_{j}^{\prime }$.

$R_{j}$ signs the navigation result using its secret key $y_{j}$. Randomly choose $r_{j}\in Z_{q}$ to compute $\sigma_{1}^{j}=g_{1}^{r_{j}}$, $\sigma_{2}^{j}=H\left(N,R_{j},e_{1i},e_{2i},e_{3i},\sigma_{1}^{j}\right)$, $\sigma_{3}^{j}=r_{j}+y_{j}\cdot \sigma_{2}^{j}$. Finally, $R_{j}$ sends the navigation result ${NR}_{j}=\left(N,R_{j},e_{1i},e_{2i},e_{3i},\sigma_{1}^{j},\sigma_{3}^{j}\right)$ to $V_{ID}^{*}$.

Upon receiving the ${NR}_{j}$, $V_{ID}^{*}$ computes $\sigma_{4}^{j}=H\left(N,R_{j},e_{1i},e_{2i},e_{3i},\sigma_{1}^{j}\right)$ and checks whether $\sigma_{1}^{j}\cdot U^{*\sigma_{4}^{j}}=g_{1}^{\sigma_{3}^{j}}$ holds. If not, $V_{ID}^{*}$ returns failure and aborts; otherwise, it decodes $OP_{j}=AES_{Dec}\left(g_{0i},e_{3i}\right)$ by computing $g_{0i}=e_{2i}e_{1i}^{-u^{*}}$.

### 4.5. Identity Revocation

Once an accepted message has been disputed, the TA can use the self-delegated certificate $Cert_{l}=\left\{U_{l},T_{1},T_{2},c,s_{\alpha },s_{v},s_{\beta }\right\}$ of $\left(V_{ID}^{*},A\right)$ to revoke the real identity of the disputed vehicle. The TA uses its secret key $\left(x_{1},x_{2}\right)\in Z_{q}^{2}$ to compute

$$T_{2}^{x_{1}}/T_{1}^{x_{2}}=A^{x_{1}}\cdot X_{3}^{x_{1}\alpha }/X_{1}^{x_{2}\alpha }=A^{x_{1}}\cdot g_{1}^{x_{1}x_{2}\alpha }/g_{1}^{x_{1}x_{2}\alpha }=A^{x_{1}}$$

and can trace the identity $V_{ID}^{*}$ by looking up the entry $\left(V_{ID}^{*},A\right)$ in the secure database.

## 5. Security Analysis

In this section, we discuss security issues of the proposed navigation scheme SPNS, i.e., authentication, confidentiality, and conditional privacy preservation.

(1) Authentication

The identity authentication of vehicles can be guaranteed by the anonymous credentials $\left(A_{1},A_{2}\right)$ issued by the TA through the system setting. For the real-time navigation query, vehicles need to generate some anonymous short-life keys $U_{l}$ by themselves, the authentication of which can also be provided by self-delegated certificates $Cert_{l}$ created by using authorized key *A*. Meanwhile, in the spatial crowdsourcing phase, crowdsourcing contributor $V_{i}^{*}$ can get the reward by showing the certificate of $U_{i}$ used in the crowdsourcing response $RP_{i}$. The identity authentication of fog nodes are also guaranteed by the certificates generated by TA.

Message authentication can be guaranteed by using randomized signatures [19] and Schnorr signatures [22]. In the real-time navigation query phase, vehicles generate signature $\left(B_{1},B_{2},C,\tau \right)$ of the navigation information by using short randomized signatures [19]. In the spatial crowdsourcing phase, vehicle $V_{i}^{*}$, who wants to perform the crowdsourcing task, returns the crowdsourcing report with a randomized signature $\left(B_{1i},B_{2i},C_{i},\tau_{i}\right)$ . In addition, when a fog node $R_{j}$ finds the optimal path $OP_{j}$, it will generate a signature $S_{j}=U^{*y_{j}}$ by using its secret key $y_{j}$ to sign the $OP_{j}$. The security of the signature depends on the discrete algorithm problem in $G_{1}$. In the navigation result retrieving phase, vehicles generate signatures of the retrieving query through randomized signatures, and fog nodes create signatures of the navigation result by using Schnorr signatures. Since both short randomized signatures and Schnorr signatures used in our scheme have proven to be unforgeable, the security of signatures generated in our proposed scheme are secure, which guarantees the message authentication.

(2) Confidentiality

To avoid navigation information being illegally obtained by unauthorized vehicles or adversaries, our scheme takes advantage of the Elgamal encryption algorithm, AES algorithm and the message-lock encryption algorithm to encrypt the transmitted information including real-time navigation query, crowdsourcing response and the navigation result. If the encryption algorithms used in the proposed scheme are secure, confidentiality requirements can be satisfied.

First, we consider the anonymous credential. When vehicle $V_{i}$ requests an anonymous credential from the TA, it first picks a random number $v\in Z_{q}$ to compute $V=g_{1}^{v}$, $V_{1}=X_{2}^{v}$, and sends $\left(V,V_{1}\right)$ to the TA along with a zero-knowledge proof to prove its knowledge of *v*. Thus, the TA cannot tell the secret key *v* of the vehicle, if the discrete algorithm problem in $G_{1}$ and $G_{2}$ is hard. Then, TA computes the anonymous credential $skv=(v,A,A_{1},A_{2})$ and sends it to the vehicle through a secure channel, so other vehicles can not illegally receive the anonymous credential by eavesdropping messages from the air.

Second, we consider the navigation query. $V_{i}$ utilizes the public key of the fog node $Y_{1}$ to encrypt $\left(U^{*},CL,DEST\right)$ by computing $C_{1}=g_{1}^{k_{1}}$, $C_{2}=g_{0}\cdot Y_{1}^{k_{1}}$, $C_{3}=AES_{Enc}(g_{0},U^{*}\left||CL||DEST\right)$ ($k_{1}\in Z_{q},g_{0}\in G_{1}$), which involves the Elgamal encryption algorithm and AES algorithm. Hence, the confidentiality of the navigation query is guaranteed. Similarly, when the fog node can not find the destination in its coverage, it will transmit the navigation query to the next fog node. The fog node will encrypt the $\left(U^{*},CL,DEST\right)$ by using the Elgamal encryption algorithm and AES algorithm. Therefore, no other vehicle can eavesdrop on the route even if they want to go to the same destination.

Third, we consider the crowdsourcing report. When a vehicle wants to perform the crowdsourcing task, it will generate a traffic information report $P_{i}$. To ensure the confidentiality of the report, $P_{i}$, we encrypt it by using the message-lock encryption algorithm RCE [15], which can ensure the confidentiality of the report and avoid the repeated rewarding for the same vehicle.

Finally, we consider the navigation result. The navigation result $OP_{j}$ is encrypted as $\left(e_{1i},e_{2i},e_{3i}\right)$ by using the public key $U_{i}$ of the querying vehicle based on Elgamal encryption. When a vehicle asks for the navigation result, it can decode the navigation result by using its short-life secret key *u*. However, other vehicles can not decrypt the ciphertext $\left(N,S_{j},e_{1i},e_{2i},e_{3i}\right)$. 

(3) Conditional privacy preservation

In our scheme, although fog nodes can decode $V_{ID}^{*}$’s short-life public key, current location, and the destination by computing $\left(U^{*},CL,DEST\right)=AES_{Dec}\left(g_{0},C_{3}\right)$, they can not link this information to some specific vehicle. Because querying vehicle $V_{ID}^{*}$ utilizes anonymous credentials $\left(A_{1},A_{2}\right)$ to prove itself. To prevent dishonest fog nodes or adversaries from linking the navigation query or the retrieving query to a specific vehicle, $V_{ID}^{*}$ provides a randomized version of the credential $\left(A_{1},A_{2}\right)$ when generating signatures. Different versions of $\left(A_{1},A_{2}\right)$ are unlinkable because linking $\left(A_{1},A_{2}\right)$ with $\left(A_{1}^{t},A_{2}^{t}\right)$ for some $t\in Z_{q}$ is equivalent to breaking the DDH assumption in $G_{1}$. Furthermore, vehicles use the group signature scheme [21] to sign messages as $\left(B_{1},B_{2},c,\tau \right)$, which provides conditional anonymity of the signer.

In addition, $V_{ID}^{*}$ takes advantage of group signatures [21] to generate short-life keys $u_{l}\left|\right|U_{l}\left|\right|Cert_{l}$ for l=1,2,⋯,m for anonymous authentication in the proposed scheme, so only TA can distinguish the real identity of $V_{ID}^{*}$. When vehicle $V_{ID}^{i}$ performs the crowdsourcing task, its anonymity and identity privacy can also be guaranteed by the randomly chosen public key $U_{i}$.

In conclusion, the anonymity, identity privacy and location privacy of the vehicles have been protected in our scheme. However, once an exceptional event occurs, the fog nodes can learn the vehicle’s real identifier with the help of TA. The TA can use the self-delegated certificate $Cert_{l}=\left\{U_{l},T_{1},T_{2},c,s_{\alpha },s_{v},s_{\beta }\right\}$ used in the navigation query to trace the identity of the disputed vehicle. Hence, conditional privacy preservation is satisfied in our scheme.

## 6. Performance Analysis

In this section, we evaluate and compare the computational and communication costs of the proposed scheme SPNS with VSPN (VANET-Based Secure and Privacy-Preserving Navigation Scheme) [23].

Firstly, let $T_{PM}$ denote the time to perform one point scalar multiplication in $G_{1}/G_{2}$, with $T_{AES}$ the time of AES encryption, $T_{par}$ the time of a pairing operation, respectively. Since these operations dominate the speed of the proposed scheme SPNS, we only consider the time taken by these operations and neglect other operations such as one-way hash function, addition and scalar value manipulation. The number of the operations required in each phase of the proposed SPNS are shown in Table 2.

Next, we consider the computational costs of TA, vehicles and fog nodes in our scheme SPNS compared with VSPN [23]. For the TA side, it is only involved in the system setting and tracing phases. The number of the operations are $8T_{PM}+2T_{par}$ and $2T_{PM}$ in SPNS, compared to $2T_{PM}$ and $2T_{PM}$ in the protocol VSPN. For the participant TA side, SPNS is less efficient than VSPN because we need more secure parameters for self-delegating short-life public keys and crowdsourcing tasks, which are not mentioned in VSPN. As shown in Table 3, for the vehicles’ side, our scheme needs more operations in the system setting phase to generate self-delegating short-life public keys, which can enhance the anonymity of the vehicles and also avoid delegating public key certificates by CA. Since our scheme accomplishes enhanced security and privacy, our scheme needs more operations than VSPN. Considering the experiment in [24] for an MNT (Miyaji, Nakabayashi, Takano) curve [25] with embedding degree k=6, G being represented by 161 bits and order *q* being represented by 160 bits, on an Intel Pentium IV 3.0-GHz machine, there exists the following results: $T_{PM}=0.6$ ms, $T_{par}=4.5$ ms. Our scheme needs 1.8 ms more to realize the navigation query than VSPN. When retrieving the navigation result from each fog node or RSU, our scheme needs 1.2 ms more than VSPN. For the fog node side, since the computational capability is stronger than common RSU in our fog-based VANET model, it is efficient to realize the operations in each phase of our SPNS scheme.

**Figure 2:**
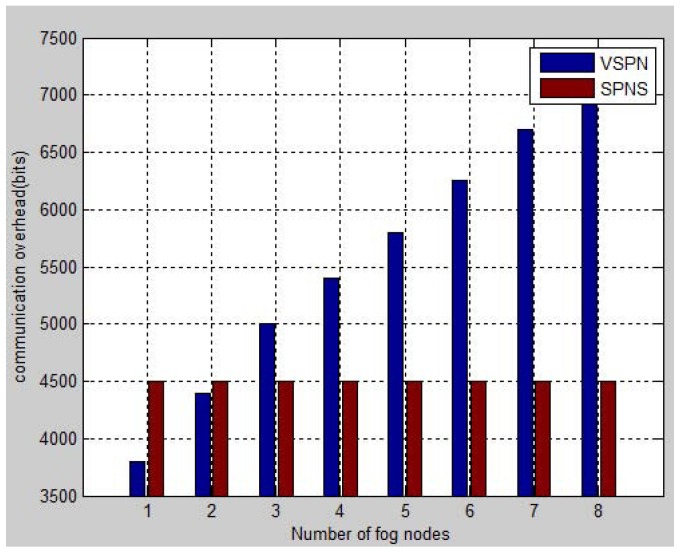
Communication overhead comparison.

In terms of the communication overhead, VSPN needs the initial RSU, $RSU_{k}$, to forward the navigation request *Q* to its neighbors until *Q* reaches the last RSU, $RSU_{d},$ covering the destination. After $RSU_{d}$ constructs the navigation reply message, it sends the message back along the reverse path to the initial RSU, $RSU_{k}$. Furthermore, the querying vehicle can get the the navigation result from the $RSU_{k}$. This procedure needs much communication among RSUs. In contrast to VSPN, our SPNS does not require the fog nodes to return the navigation results to the first fog node. Instead, the querying vehicle can retrieve the navigation result from each fog node and use it to find a proper route to the destination or to the next fog node. In this way, the communication overhead among fog nodes is significantly reduced. Figure 2 shows the comparison results of SPNS and VSPN with respect to the average communication burden between two fog nodes.

## 7. Related Works

A number of previous studies have been dedicated to designing real-time VANET-based navigation systems for the last few years. In 2009, Lu [26] presented a VANET-based parking navigation protocol, which tracks available parking spaces and guides drivers to the available parking spaces. In their protocol, three RSUs are fully trusted, which provide the navigation functions for a vehicle to find a vacant parking space in a parking lot. However, the protocol [26] can not be used for our navigation purposes. In 2010, another work [27] gave an application of real-time navigation. In addition to driving guidance, the returned routes of the scheme [27] were used for opportunistically routing multimedia information such as images and videos of a desired scene to vehicles. In addition, VANET-based navigation systems [28,29] also emerged to provide real-time navigation services for drivers on roads. By means of the widely deployed vehicular communication infrastructure, the vehicles only needed the OBUs to enjoy the navigation services. However, the security and privacy issues were not concerned in their schemes.

Recently, several VANET-based vehicle navigation systems [23,30,31,32] have been proposed for drivers’ privacy preservation. Chim et al. [23] proposed a VANET-based secure and privacy-preserving navigation system, which utilizes the anonymous credentials to provide secure navigation services to drivers. Based on anonymous credentials and the destination, the system can use the real-time road information to search for an available route for drivers in a distributed way. Nevertheless, this system is vulnerable to insider attacks since the system master key is shared among all vehicles. To eliminate the system master key distribution and simplify the anonymous credential acquisition, Cho et al. [30] introduced a security-enhanced navigation system based on the concept of two person multisignature [33] and identity-based cryptographic schemes [34]. However, how to collect traffic information was not considered in their scheme. Sur et al. [31] pointed out that prior VANET-based secure navigation protocols cannot provide non transferability of anonymous credentials used in their protocols to prevent an insider attacker from sharing her anonymous credentials, and the protocols [23,30] are vulnerable to an attacker who can compromise roadside units (RSUs) deployed on the roads. Sur et al. [31] proposed a secure navigation system based on vehicular cloud from a trapdoor hash function and zero-knowledge proof. However, the anonymous credentials in this system can only be used once for fear of vehicles’ sharing credentials with unregistered users. Ni et al. [32] proposed a privacy-preserving real-time navigation system using crowdsourcing. However, their scheme does not take advantage of fog computing, which leads to high efficiency and low-latency.

Our scheme is based on the idea of randomizing anonymous credentials. Once the fog nodes are compromised, they can not link the navigation query or a retrieving query to a specific vehicle. In this way, it can preserve the privacy of the vehicles. Moreover, the anonymous credentials need not be updated frequently, whereas it can be used for a long time. In our scheme, we utilize fog nodes to issue spatial crowdsourcing tasks to vehicles in their coverage in order to collect real-time road conditions, which guarantee that the retrieving path is real-time and optimal. In addition, the querying vehicle can successively retrieve the navigation result from each fog node when entering its coverage area. This framework is superior to existing solutions, which mainly depend on the assumption that a moving vehicle has to obtain the results from the first fog node, which is quite challenging in reality due to vehicles’ high moving speed.

## 8. Conclusions

In this paper, we proposed a secure and privacy-preserving real-time navigation system based on fog-based VANETs. We utilized the real-time traffic information to guid the vehicle to a desired destination in a distributed way: fog nodes generate the spatial crowdsourcing task to collect real-time road conditions. Then, each fog node takes advantage of the collected traffic information provided by the vehicles in its coverage to compute the optimal route to the destination. Vehicles can get the continuous optimal route from the fog nodes until it arrives at the desired destination. Moreover, the vehicle performing the crowdsourcing task can get a reasonable reward. Our scheme adopts some security primitives to provide a number of security features: (1) vehicles are authenticated by using zero-knowledge proof and randomized anonymous credentials; (2) messages provided by the vehicles and fog nodes can also be authenticated by means of signatures; (3) navigation queries, traffic information report and navigation results are protected from eavesdroppers. Besides satisfying all security requirements, our scheme provides the conditional privacy-preserving requirements. No one including TA can link up a vehicle’s navigation query and its identity. However, the TA can trace the identity of the driver who reports false traffic information. Furthermore, our scheme is efficient in terms of computational and communication overhead. For the future work, we will further improve the effectiveness of our scheme and develop a privacy-preserving parking system using vehicular crowdsourcing based on fog-based VANETs.

## Figures and Tables

**Table 1:** The description of navigation information elements.

Element	Description
*N*	Sequence number: records the query number that is used to distinguish different queries from the same OBU.
$U^{*}$	Short-life public key: If a vehicle sends a navigation query at some time, it will randomly choose a short-life public key $U^{*}$ from the sequence $u_{l}\left|\right|U_{l}\left|\right|Cert_{l}$ for l=1,2,⋯,m stored in the OBU. This field is used to record the public key, which will be also used to reward vehicles in the spatial crowdsourcing step.
CL	Current location: records the current position of the querying vehicle on the unique Euclidean plane.
DEST	Desired destination: records the destination where the querying vehicle will arrive.
$t_{c}$	Current time: records the start querying time.
$t_{e}$	Expired time: records the exact time after which the query is invalid, because the life-time of the navigation query is fixed.

**Table 2:** Computational cost of each step in SPNS.

Phases	TA	Fog Node	Vehicle
System setting	$8T_{PM}+2T_{par}$	$T_{PM}$	$\left(2+m\right)T_{PM}+2mT_{par}$
Querying	0	$6T_{PM}+3T_{par}+2T_{AES}$	$4T_{PM}+T_{AES}+T_{par}$
Crowdsourcing	0	$6T_{PM}+3T_{par}+2T_{AES}$	$2T_{PM}+T_{AES}$
Retrieving	0	$4T_{PM}+3T_{par}+2T_{AES}$	$n(6T_{PM}+T_{par}+T_{AES})$
Tracing	$2T_{PM}$	0	0

*m* is the number of the short-life public keys generated by vehicles and *n* is the number of the fog nodes that relay the navigation query; SPNS is our proposed scheme.

**Table 3:** Comparison of vehicles’ computational cost.

Phases	SPNS	VSPN
Setting	$\left(2+m\right)T_{PM}+2mT_{par}$	$9T_{PM}+T_{par}+2T_{AES}$
Querying	$4T_{PM}+T_{AES}+T_{par}$	$T_{PM}+T_{AES}$
Crowdsourcing	$2T_{PM}+T_{AES}$	0
Retrieving	$n(6T_{PM}+T_{par}+T_{AES})$	$4nT_{PM}$

*m* is the number of the short-life public keys generated by vehicles and *n* is the number of the fog nodes that relay the navigation query; SPNS is our proposed scheme; VSPN is the VANET-Based Secure and Privacy-Preserving Navigation Scheme proposed in [23].
